# Editor Responsibility and Scientific Integrity During the COVID-19 Outbreak

**DOI:** 10.4274/balkanmedj.galenos.2020.2020.4.001

**Published:** 2020-06-01

**Authors:** Zafer Koçak, Cem Uzun

**Affiliations:** 1 Department of Radiation Oncology, Trakya University School of Medicine, Edirne, Turkey; 2 Department of Otolaryngology, Trakya University School of Medicine, Edirne, Turkey

The COVID-19 outbreak has led to a speedy publication of many academic articles about the new coronavirus. Scientists and editors are under immense pressure to share their findings quickly because of the pandemic. These circumstances are bound to affect the scientific integrity adversely if the publishing activities do not comply with the universal standards and guidelines.

The Reuters’ analysis of three preprint servers BioRxiv, MedRxiv, and ChemRxiv on February 19 pointed out that “at least 153 studies have been posted or published, including epidemiological documents, genetic analyses, and clinical reports that have examined every aspect of the disease since the outbreak began” (1). However, during the 2003 SARS epidemic, even half this number of studies on SARS issue took over a year to be published. Another analysis showed us that in just 2.5 months, more than 3000 articles about COVID-19 were accepted by journals (2).

For example, a study by Indian scientists reported “mysterious” similarities between the new coronavirus and HIV. The study was criticized by scientists around the world and hence it was quickly withdrawn (3). There was another clinical study on the treatment of COVID-19 infections with hydroxychloroquine and azithromycin that was accepted through a one-day peer review process (4). More retracted or withdrawn articles on COVID-19 can be found on the "retracted watch" website (5).

Therefore, it is alarming to observe some editorial practices that ignore the ethical principles and this will affect the scientific integrity of the papers published during this period. Recently, European Association of Science Editors (EASE) made a statement about quality standards in publishing (6). EASE encourages anyone involved in the collection and publication of pandemic data to comply with the ethical guidelines and follow standard reporting guidelines, such as CONSORT for clinical trials, STROBE for epidemiological studies, and SAGER guidelines.

From the first week of February, we started receiving manuscripts about COVID-19. By the way, it was really strange to realize that we have not received any articles about corona virus in recent years.As the editorial staff, we decided to check our attitude toward COVID-19 articles. In the period February 2020-March 2020, a total of 18 studies on COVID-19 were submitted to our journal (Table 1). Almost half of these studies were from China. In total, 16% of these articles were original articles, and all of them were rejected after editorial review. Four (22%) out of the total 18 studies were accepted. The median times for acceptance and rejection were 3.5 and 9 days, respectively.

Compared to the prepandemic period, we observed a significant reduction in the time required for acceptance and rejection. For the year of 2019, the average acceptance and rejection times were 85 and 21 days, respectively. One of the factors that led to this significantly shortened article acceptance time was that these articles were evaluated not through peer review processes, but were reviewed by the editorial board that includes experts in the field of the submitted manuscripts. Another factor was the desire to quickly provide trustful data about the pandemic to the scientific world.

In conclusion, the COVID-19 pandemic has been showing us how important it is to edit and distribute accurate data via scientific journals. All the publishing staff has a responsibility and should take enormous efforts to provide quick but correct information, which can be guaranteed only by following the international publishing standards.

## Figures and Tables

**Table 1:**
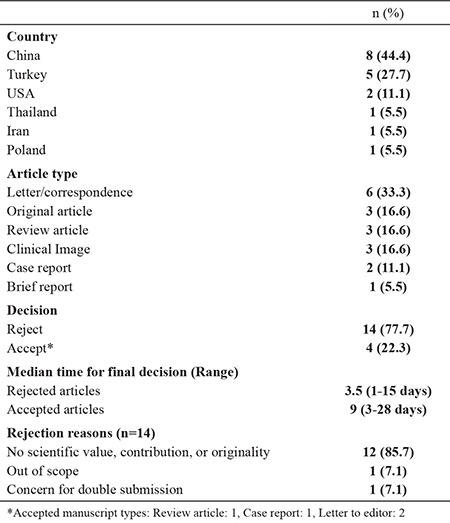
Articles on COVID-19 submitted to Balkan Medical Journal from February 2, 2020 to April 7, 2020
